# Bibliographical Notices

**Published:** 1847-09

**Authors:** 


### BIBLIOGRAPHICAL NOTICES.

*Saint Louis Medical and Surgical Journal.*—This Journal, which has hitherto been a monthly of forty-eight pages, commences its fifth volume in an improved dress, and is hereafter to be issued bi-monthly, containing one hundred and four pages, at three dollars per annum. It continues under the editorial charge of Drs. Linton and McPheeters. It is an able, spirited journal; and we cordially congratulate its accomplished editors upon its increasing popularity.

*Ranking's Half-yearly Abstract of the Medical Sciences.*.—The first number of volume third of this excellent periodical has been promptly reprinted by Lindsay & Blakiston, of Philadelphia. In addition to the selected articles, it contains reports on Practical Medicine, by the Editor; on Surgery, by Henry Ancell, Esquire; on Midwifery and Diseases of Women and Children, by the Editor; on Chemistry, by George Edward Day, M. D.; on Forensic Medcine, by William Augustus Guy, M. D.; and on the Ether Inhalation, by the Editor. These reports are able papers, presenting a careful summary of the various contributions which have been made for the preceding six months toward the progress of the departments of Medical Science to which they respectively relate. The work is deservedly much esteemed by the profession.

---

*A Treatise on the Practice of Medicine.* By GEORGE B. WOOD, M. D., Professor of Materia Medica and Pharmacy in the University of Pennsylvania, &c.; in two volumes, 8vo., Philadelphia, Grigg, Elliott & Co., No. 9 North Fourth St., 1847.

The author of this work, our readers need not be informed, occupies a distinguished position among the Medical *savans* of this country. Author, in conjunction with Dr. Bache, of the United States Dispensatory, eminent as a teacher of Materia Medica, a practitioner of large and long experience, and distinguished for his general scholastic and scientific acquirements, the expectations of the profession would hardly have been satisfied with a production of mediocre merit emanating from so high a quarter. We take pleasure in expressing our conviction, that great as must be the anticipations of the character of the work, they will not be disappointed. We do not hesitate to say, that, in our opinion, Dr. Wood has given us a work on Practical Medicine which is not excelled by any other in the English language. We do not, of course, mean to affirm that it is without faults, but we can conscientiously say that, in our judgment, it has fewer defects than any other work of a similar character with which we are acquainted. We know of no work on Practice which presents a more comprehensive and discriminating outline of Practical Medicine, in the present stage of its progress, than this. We might particularize the several points of view in which it appears to us to merit these encomiums; but this is not necessary, since they who may in any degree be influenced by our opinion, will probably obtain the work, and judge for themselves of its various excellencies, as well as its faults. Suffice it say, that its pathology is based upon a rational eclecticism, its description of symptoms is full and lucid; its therapeutics prudent but decided, effective but judicious, and the arrangement and execution of the work exhibit philosophical reflection, sound judgment, and an agreeable style of composition. We pen these remarks after a general examination of the whole work, and a careful perusal of some parts of it. We may, hereafter, venture upon a critical analysis of certain portions, when we shall not hesitate, with all candour and frankness, to indulge such strictures as seem to us to be called for; but, as a whole we can only speak of it in terms of unqualified praise. As an American production we congratulate the profession upon so valuable a contribution to our native medical literature, and we predict for it all the success which the author and his friends could reasonably desire.

